# Face masks vs. COVID-19: a systematic review

**DOI:** 10.17533/udea.iee.v38n2e13

**Published:** 2020-07-10

**Authors:** Behzad Fouladi Dehaghi, Abbas Ghodrati-Torbati, Gholamheidar Teimori, Leila Ibrahimi Ghavamabadi, Amir Jamshidnezhad

**Affiliations:** 1 Associate Professor, Environmental Technologies Research Center, Ahvaz Jundishapur University of Medical Sciences and Department of Occupational Health, School of Public Health, Ahvaz Jundishapur University of Medical Sciences, Ahvaz, Iran. Email: bdehaghi@gmail.com Ahvaz Jundishapur University of Medical Sciences Ahvaz Jundishapur University of Medical Sciences Iran bdehaghi@gmail.com; 2 Ph.D. Department of Nursing, School of Nursing and Midwifery, Torbat Heydariyeh University of Medical Sciences, Torbat Heydariyeh, Iran. Email: ghodratita171@yahoo.com Torbat Heydarieh University Torbat Heydariyeh University of Medical Sciences Iran ghodratita171@yahoo.com; 3 M.Sc. Department of Occupational Health Engineering, School of Health, Torbat Heydariyeh University of Medical Sciences, Torbat Heydariyeh, Iran. Email: teimorigh1@gmail.com Torbat Heydariyeh University of Medical Sciences Iran teimorigh1@gmail.com; 4 Health Sciences Research Center, Torbat Heydariyeh University of Medical Sciences, Torbat Heydariyeh, Iran. Torbat Heydarieh University Torbat Heydariyeh University of Medical Sciences Iran; 5 Ph.D. Department of Environmental management-HSE, Ahvaz Branch, Islamic Azad University. Ahvaz, Iran. Email:ebrahimi.ghavam@gmail.com. Corresponding Author Islamic Azad University Islamic Azad University Iran ebrahimi.ghavam@gmail.com; 6 Ph.D. Department of Health Information Technology, Faculty of Allied Medical Sciences, Ahvaz Jundishapur University of Medical Sciences, Ahvaz, Iran. Email: dr.jamshidnejad@gmail.com Ahvaz Jundishapur University of Medical Sciences Ahvaz Jundishapur University of Medical Sciences Iran dr.jamshidnejad@gmail.com

**Keywords:** COVID-19, coronavirus infections, masks., COVID-19, infecciones por coronavirus, máscaras., COVID-19, infecções por coronavirus, máscaras.

## Abstract

The coronavirus disease (COVID-19) spread rapidly around the world. Two types of approaches have been applied to use of face masks as a tool to prevent the spread this disease in society. The aim of the systematic review was to assess the effectiveness of face masks against the novel coronavirus. A literature search was performed using different databases until April 30, 2020. Search terms were ‘facemasks’, ‘novel coronavirus’, and ‘healthcare workers’. Five studies were included in the systematic review. A study stated that no difference between surgical and cotton masks. Also, two studies have emphasized the use of surgical masks or N95 respirators by medical staff, and two other studies emphasized the use of any type of face mask by general public. More studies in controlled contexts and studies of infections in healthcare and community places are needed for better definition of the effectiveness of face masks in preventing coronavirus.

## Resumen

La enfermedad por coronavirus (COVID-19) se propagó rápidamente por todo el mundo. Se han aplicado dos tipos de enfoques al uso de máscaras faciales como herramienta para prevenir la propagación de la enfermedad en la sociedad. El objetivo de la revisión sistemática fue evaluar la efectividad de las máscaras faciales contra el nuevo coronavirus. Se realizó una búsqueda bibliográfica utilizando diferentes bases de datos hasta el 30 de abril de 2020. Los términos de búsqueda fueron: ‘máscaras faciales’, ‘nuevo coronavirus’ y ‘trabajadores de la salud’. Se incluyeron cinco estudios en la revisión sistemática. Un estudio indicó que no hay diferencia entre las máscaras quirúrgicas y las de algodón. Además, dos estudios han enfatizado el uso de máscaras quirúrgicas o respiradores N95 por parte del personal médico, y otros dos estudios enfatizaron el uso de cualquier tipo de mascarilla por parte del público en general. Se necesitan más estudios en contextos controlados y estudios de infecciones en el cuidado de la salud y en lugares comunitarios para una mejor aclaración de la efectividad de las mascarillas para prevenir el coronavirus.

## Resumo

A doença de coronavírus (COVID-19) se espalhou rapidamente por todo o mundo. Dois tipos de abordagens foram aplicados ao uso de máscaras faciais como uma ferramenta para impedir a propagação da doença na sociedade. O objetivo da revisão sistemática foi avaliar a eficácia das máscaras faciais contra o novo coronavírus. Uma pesquisa bibliográfica foi realizada usando diferentes bancos de dados até 30 de abril de 2020. Os termos de pesquisa foram: máscaras faciais ',' novo coronavírus 'e' profissionais de saúde '. Cinco estudos foram incluídos na revisão sistemática. Um estudo indicou que não há diferença entre máscaras cirúrgicas e máscaras de algodão. Além disso, dois estudos enfatizaram o uso de máscaras cirúrgicas ou respiradores N95 pelo pessoal médico e dois outros estudos enfatizaram o uso de qualquer tipo de máscara pelo público em geral. É necessário mais estudos em ambientes controlados e estudos de infecções nos serviços de saúde e na comunidade para esclarecer melhor a eficácia das máscaras na prevenção do coronavírus

## Introduction

The coronavirus (COVID-19) epidemic broke out in 2020 in Wuhan, China, and spread rapidly around the world. The severity of the disease now appears to be more severe than originally estimated.(1,2) Individual intervention approaches include improving personal hygiene (regular hand washing), wearing disposable gloves and using a face mask.(3) According to the general guidelines of the British Columbia Disease Control and Prevention Centers in Canada, the use of face masks is only recommended for sick people.(4) This inconsistency also applies to acute respiratory distress syndrome (SARS) and epidemic flu. The World Health Organization, the use of face masks recommends in low-risk conditions and respirators in high-risk conditions, but the Centers for Disease Control and Prevention (CDC) suggests the use of respirators in both low-risk and high-risk conditions.(5) The purpose of this systematic review was to investigate the effectiveness of face masks against respiratory infections, including coronavirus.

## Methods

This systematics review was conducted by matching the guidelines provided by the PRISMA declaration. A survey of articles published up to 30 April 2020 about the effectiveness of face masks against coronavirus infections was performed using four electronic databases: PubMed, Scopus, Science Citation Index (Web of Science), and Google scholar. The following terms were used in the search strategy: [‘Facemask’ OR ‘Facemasks’ OR ‘Mask’ OR ‘Masks’ OR ‘Respirator’ OR ‘Respirators’] AND [‘COVID-19’ OR ‘Coronavirus’ OR ‘Novel Coronavirus’] AND [‘Medical staff’ OR ‘Healthcare staff’ OR ‘health care providers’ OR ‘Healthcare providers’ OR ‘Healthcare workers’ OR ‘Health workers’ OR ‘Healthcare Professionals’].

Two researchers independently examined the titles and abstracts of all articles for potential liability in this review. They then evaluated the full articles to be included in the study. In case of disagreement, further study and evaluation with other authors was used to resolve data mining differences. Also, systematic review articles were excluded. But the references lists were searched for relevant papers. Further, a manual search was carried out with the first authors’ reference database.

**Figure 1:**
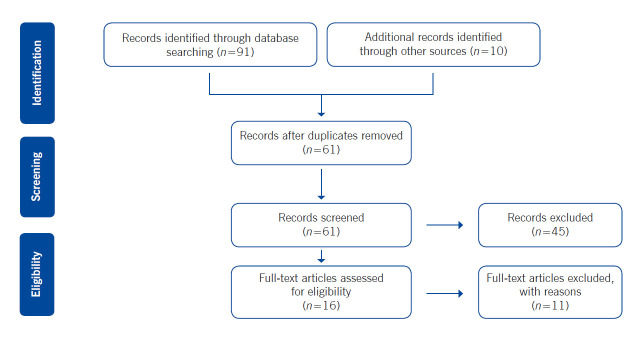
Flow diagram identifying relevant studies

Articles were included in the study which; 1- qualified controlled volunteer studies of coronavirus filtration of respirators or face masks, 2- qualified observational or intervention studies of respirators or face masks to prevent coronavirus (COVID-19) in community settings or healthcare settings. The primary search provided 101 citations. Of these, 61 studies were selected based on titles and abstracts. Then excluding irrelevant studies (*n*=41), 16 articles were accepted full-text review and were discerned for eligibility. The reasons of the excluded studies were as follows: editorials, Meta-analysis, systematic reviews and they were not written in the English language. Finally, five articles were considered relevant for inclusion in the study (Fig. 1).

## Results

The surveyed studies were significantly different in terms of design, participants, interventions and actions. So this study focused on the description of studies, results, their application and limitations in qualitative composition, not on the meta-analysis (Table 1). Bae et al. (2020) examined the efficacy of surgical and cotton face masks in filtering SARS-CoV-2. They tested the performance of disposable surgical and reusable cotton masks to filter the virus in 4 participants, with confirmed coronavirus infection.(6) Patients coughed 5 times onto a Petri dish containing 1mL of viral transport medium held nearly 20 cm in front of participants' mouth. In four stages that were as follows: wearing no masks, surgical mask, cotton mask, and again with no mask. Also, both outer and inner surfaces of masks were swabbed with aseptic Dacron swabs. Coronavirus could be detected on the Petri dish specimens when participants coughed without a mask (in 4 subjects), coughing with a surgical mask (in 3 subjects) and coughing with a cotton mask (in 2 subjects). Also, all swabs from the outer surgical and cotton mask surfaces were positive for SARS-CoV-2, and most swabs from the inner mask surfaces were negative. Limitations were that the study did not consider included other face masks as N95 and the role of air penetration around the borders of the mask.(6) In a retrospective study by Wang *et al*., the disease-related data ranged from January 2 to January 22, 2020, in six different wards (lung, ICU, infectious, pancreatic liver surgery, trauma, microsurgery and urology) from Zhongnan Hospital at Wuhan University, China. Health care workers from respiratory, infectious diseases and ICU wards which used N95 respirators, disinfectants, and cleaned hands frequently, entered the study as the "N95 group". Due to the lack of knowledge about COVID-19 in the early days of the outbreak, medical personnel in all other three wards of the hospital did not use any medical masks and occasionally used disinfectants and hand sanitizers. The group was considered a "without mask group". Suspected cases of COVID-19 infection were diagnosed with CT of the chest and confirmed by molecular diagnostic methods. Of the total patients, 28 confirmed and 58 suspected cases were identified during the data collection period. 

**Table 1:** Studies conducted in healthcare settings

Study	Setting	Mask type	Findings
Bae *et al*.,^(6)^	2 hospitals, Seoul, 2020	Surgical & cotton masks	No difference between surgical and cotton masks
Wang *et al.,* ^*(7)*^	A hospital, Wuhan, 2020	N95	emphasize use the N95 respirator by health care workers
Chang *et al*.,^(8)^	43 public hospitals, Hong Kong, 2020	Surgical mask	Attention to the principles of infection prevention in the hospital and use the surgical mask
Eikenberry *et al*.,^(9)^	A compartmental model	N95, Surgical & cloth masks	Use of face masks by the general public
Worby and Chang^(10)^	Epidemic models	Face masks	Face mask use

The medical staff's contact with COVID-19 patients in the N95 group was significantly higher than the group without mask. According to the results, it was revealed that out of 493 people in the N95 group which was consisted of 278 (222 nurses and 56 physicians), no one was infected by COVID- 19 disease. And in masked groups (136 nurses and 77 doctors), 10 were infected.(7) Chang *et al.* studied the preparation for infection control for coronavirus (COVID-19) due to SARS-CoV-2 in the first 42 days after the proclamation of pneumonia in China. Therefore, environmental samples and air samples were collected and analyzed. The RNA of virus was not detected in 8 air samples collected in a 10 cm distance from the patient's chin. It has been suggested that the virus is not transmitted through the airways, which is not reliable based on a patient's analysis. This can also be due to the rapid dilution of air inside the room separating airborne infection or airway. It was found that from day one to day 42 of the 1275 patients had positive test results of SARS-CoV-2 infection. Of the 413 health care workers confirmed, 11 (2.7%) were exposed to unprotected and quarantine for 14 days. However, no COVID-19 hospital transfers were observed and appropriate measures to control nosocomial infections were able to prevent SARS-CoV-2 nosocomial transmission.(8) A modelling study by Eikenberry *et al.*(9) suggested that use of face masks should be performed by the general public as much as possible and without delay all around the world. Even if most of the masks are home-made and of relatively low quality. These measures could be of great help in controlling the COVID-19 pandemic, along with other non-pharmacological interventions that reduce community transmission. Another modelling study by Worby and Chang(10) found that face masks, even with limited protective properties, can reduce infections and death rates, and can delay the onset of the disease.(10) Therefore, the use of face masks, especially for a disease with asymptomatic conditions, is relatively common and can effectively reduce its spread.

## Discussion

The present review study emphasizes on limited evidence to support the effectiveness of the face masks to reduce the transmission of the Coronavirus. An important concern when determining which public health intervention can be helpful in reducing the Coronavirus epidemic and which methods of infection control are essential to prevent the transmission of the disease, it is vital to know how the Coronavirus is transmitted between the individuals and the environment. It is recommended to use medical and fabric masks to prevent contamination of the healthcare workers.(7,9) People who do not have respiratory symptoms do not need to wear the N95 respirators, even if COVID-19 is prevalent in the area. However, the use of surgical and cotton masks in crowded environments (such as public transport) is recommended for high-risk individuals (the elderly, pregnant women, and people with underlying diseases), and it is important to note this. Hand contact with the outer layer of the mask should be avoided due to the accumulation of contamination.(6) The use of masks does not diminish the importance of other general measures to prevent infections.(6,7) 

## Conclusion.

There is little evidence to support the effectiveness of face masks to reduce the risk of COVID-19 infection. However, the use of N95 respirators or air supplying respirators and adherence to the principles of personal hygiene, frequent hand washing and the use of disinfectants can reduce the prevalence of COVID-19 in health care providers. Due to the novelty of the COVID-19 virus, no clinical trials have been found on the use of face masks in disease prevention. Also, the use of face masks by people in the community, in addition to other health principles can help in reducing the prevalence of COVID-19 disease.
